# Quantitative proteomic analysis reveals potential diagnostic markers and pathways involved in pathogenesis of renal cell carcinoma

**DOI:** 10.18632/oncotarget.1529

**Published:** 2014-01-18

**Authors:** Nicole M.A. White, Olena Masui, Leroi V. DeSouza, Olga Krakovska-Yutz, Shereen Metias, Alexander D. Romaschin, R. John Honey, Robert Stewart, Kenneth Pace, Jason Lee, Michael AS Jewett, Georg A. Bjarnason, K.W. Michael Siu, George M. Yousef

**Affiliations:** ^1^ The Keenan Research Center in the Li Ka Shing Knowledge Institute and the Department of Laboratory Medicine, St. Michael's Hospital, Toronto, Canada; ^2^ Department of Laboratory Medicine and Pathobiology, University of Toronto, Toronto, Canada; ^3^ Department of Chemistry and Centre for Research in Mass Spectrometry, York University, Toronto, Canada; ^4^ Department of Surgery, St. Michael's Hospital, Toronto, Canada; ^5^ Division of Urologic Oncology, Princess Margaret Hospital, University Health Network, Department of Surgery, University of Toronto, Toronto, ON, Canada; ^6^ Division of Medical Oncology and Hematology, Sunnybrook Health Sciences, Toronto, Canada

**Keywords:** kidney cancer, renal cell, carcinoma, iTRAQ, proteomics, Hsp27, urine markers

## Abstract

There are no serum biomarkers for the accurate diagnosis of clear cell renal cell carcinoma (ccRCC). Diagnosis and decision of nephrectomy rely on imaging which is not always accurate. Non-invasive diagnostic biomarkers are urgently required. In this study, we preformed quantitative proteomics analysis on a total of 199 patients including 30 matched pairs of normal kidney and ccRCC using isobaric tags for relative and absolute quantitation (iTRAQ) labeling and LC-MS/MS analysis to identify differentially expressed proteins. We found 55 proteins significantly dysregulated in ccRCC compared to normal kidney tissue. 54 were previously reported to play a role in carcinogenesis, and 39 are secreted proteins. Dysregulation of alpha-enolase (ENO1), L-lactate dehydrogenase A chain (LDHA), heat shock protein beta-1 (HSPB1/Hsp27), and 10 kDa heat shock protein, mitochondrial (HSPE1) was confirmed in two independent sets of patients by western blot and immunohistochemistry. Pathway analysis, validated by PCR, showed glucose metabolism is altered in ccRCC compared to normal kidney tissue. In addition, we examined the utility of Hsp27 as biomarker in serum and urine. In ccRCC patients, Hsp27 was elevated in the urine and serum and high serum Hsp27 was associated with high grade (Grade 3–4) tumors. These data together identify potential diagnostic biomarkers for ccRCC and shed new light on the molecular mechanisms that are dysregulated and contribute to the pathogenesis of ccRCC. Hsp27 is a promising diagnostic marker for ccRCC although further large-scale studies are required. Also, molecular profiling may help pave the road to the discovery of new therapies.

## INTRODUCTION

Renal cell carcinoma (RCC) is the most common neoplasm in the adult kidney, with an increasing incidence over the past 20 years[1]. About 80% of RCCs are of the clear-cell type (ccRCC). Early diagnosis of RCC is associated with favorable prognosis (5-year survival rate ~ 85%). Unfortunately, RCC is often asymptotic, with about 30% of patients diagnosed at the metastatic stage when the prospects for cure are dismal (5-year survival rate ~9%) [2]. Traditional manifestations of pain, mass and hematuria are ineffective for early diagnosis [3]. The diagnosis of RCC, and the subsequent resection of the kidney are based on imaging findings, which are not always accurate. There are currently no serum biomarkers available to confirm the identity of renal masses, whether benign or malignant. A non-invasive test in serum or urine will have a significant impact on patient management.

Few chromosomal abnormalities have been documented in RCC, including VHL mutation (3p-), 5q21+ (70%), and 14q- (41%)[4;5]. The pathogenesis of RCC is, however, not yet fully elucidated. Understanding tumor biology at the molecular level is essential in order to improve treatment[6;7].

Proteomics combined with mass spectrometry (MS) offers great promise for unveiling the complex molecular events of tumorigenesis and identification of cancer biomarkers. Tissue proteomics is a promising alternative strategy for the discovery and identification of tumor markers. A major advantage of tissues proteomics is that relevant proteins are much more abundant. Additionally, the link between proteins that are differentially expressed in diseased tissue with the disease itself is stronger than serum.

In this study, we performed quantitative proteomic analysis using isobaric tags for relative and absolute quantitation (iTRAQ) labeling and LC-MS to identify proteins that are dysregulated in ccRCC compared to normal kidney. We identified a number of proteins that can distinguish between tumor and normal tissues. We verified the dysregulated expression of four of the most interesting proteins on two independent sets of tissue using both western blot and immunohistochemistry. Also, these proteins showed to have the ability to distinguish between normal and cancer tissue with accuracy. Identified secreted proteins may serve as diagnostic makers. Furthermore, using pathway analysis and protein-protein analysis, we elucidated the potential involvement of these proteins in RCC pathogenesis and identified key pathways that are dysregulated in RCC. We also examined the use of Hsp27 as a biomarker in the serum and urine and its prognostic utility.

## RESULTS

### Identification of dysregulated proteins between ccRCC and normal tissues

A schematic of the work flow is shown in Supplementary Figure 1. A total of 40 samples were analyzed in the discovery phase. Briefly, equal amounts of protein from each tissue type were digested with trypsin, labeled with iTRAQ and combined. Samples were then separated by off-line SCX liquid chromatography and analyzed by RP LC-MS. We identified a total of 1591 non-redundant proteins with local false discovery rate (FDR) < 5% (Supplementary Table 5); 345 of these proteins were reliably quantified (Supplementary Table 6). Fifty five proteins fulfilled our criteria for dysregulation between ccRCC and normal (see materials section, Supplementary Table 7): 15 were upregulated (iTRAQ ratios of ≥1.5) and 40 were downregulated (iTRAQ ratios of ≤0.67). Table 1 shows a heat map of the 55 significantly dysregulated proteins.

**Table 1:** A list of 55 proteins significantly dysregulated in ccRCC compared to normal kidney tissue samples as identified by quantitative proteomic analysis

Protein Symbol	C1	C2	C3	C4	C5	C7	C8	C9	C10	C6	N1	N2	N3	N4	N5	N6	N7	N8	N9	N10
AHNAK	1.52	1.73	1.40	1.36	1.12	1.73	1.35	1.66	1.58	0.84	0.90	0.89	0.89	0.99	1.18	0.82	1.21	1.00	1.05	1.23
ENO1	1.94	2.69	1.86	2.05	1.45	1.87	1.62	1.94	2.35	0.94	1.07	0.74	0.90	0.89	1.11	0.79	1.18	1.01	1.07	0.91
HSPB1	2.30	3.51	2.14	2.24	1.37	2.92	1.53	3.01	2.33	0.81	1.14	0.88	1.11	0.98	0.69	0.83	1.29	0.64	1.20	1.09
LDHA	1.94	2.64	2.97	3.60	2.55	4.93	3.92	3.20	4.12	0.78	0.53	1.07	0.80	1.03	0.97	0.63	1.13	0.99	1.04	0.97
ALDOA	1.72	1.51	1.16	1.15	1.33	2.36	1.46	1.74	1.87	0.82	0.81	0.73	0.87	0.98	1.21	0.71	1.08	0.95	1.00	1.09
ANXA2		2.41	2.16	2.23	1.31	1.59	2.80		1.93	0.95		1.11	0.98	0.73	1.22	1.04				
ANXA4	3.19	3.27	3.80	1.95	2.11	5.79	3.05	5.44	3.70	0.96		0.66	0.92	0.96	0.82	0.66	0.64	0.83	0.98	0.96
ANXA5	1.62	1.82	1.52	2.36	1.12	1.83	1.73	1.60	2.27	0.82	0.84	0.99	0.88	0.94	1.38	0.68	1.15	0.97	1.05	0.95
CNDP2	1.53	1.56	2.01	1.42	1.34	2.30	1.30	2.51	1.91	1.01	1.11	0.90	0.93	1.02	0.90	0.86	0.93	0.94	1.09	0.97
CRYAB	2.20	3.83	2.34	1.84	0.90	3.65	1.33	2.59	1.99		1.59	0.53	1.42	0.85	0.56	0.50	1.27	0.57	1.12	1.24
GAPDH	1.37	1.68	1.67	1.45	1.10	1.89	1.83	1.91	2.12	0.87	0.96	0.83	0.93	0.90	0.77	0.72	0.98	0.87	0.95	0.95
MIF	2.07	2.71	3.97	2.30		2.47	1.69	2.32			1.23	1.54		1.04	1.14	0.82	0.92	0.96	0.90	
PGK1	1.93	2.60	2.14	1.68	1.34	1.52	1.47	1.63	2.21	1.02	0.89	0.91	0.98	1.00	1.02	0.81	1.04	0.94	0.99	1.11
PKM2	2.73	3.47			2.38		2.88	4.18	1.73	0.78	0.97		0.70			0.63	1.12	0.87	0.98	
TPI1	1.69	2.11	1.54	1.44	1.41	2.10	1.69	2.17	1.68	0.97	0.99	0.78	0.93	1.05	1.04	0.87	1.11	0.93	0.99	0.95
HSPE1	0.72	0.54	0.45	0.22		0.50	0.50	0.51	0.44	1.21	1.31	0.90	1.25	0.93	0.85	0.96	1.19	0.94	0.89	0.97
ACAA2	0.70	0.54	0.73	0.37	0.31	0.65	0.39	0.51	1.07	1.23	0.95	1.16	1.24	0.94	0.68	0.84	0.97	1.05	0.90	1.33
ACADM	0.34	0.34	0.43	0.28	0.46	0.44	0.56	0.37	0.59	1.27	1.15	0.91	1.18	0.93	0.73	1.05	1.20	0.96	0.95	1.00
ACAT1		0.68	0.51	0.24		0.33	0.33	0.34	0.50	1.19	1.21	1.35	1.14	0.96	0.76	0.96	0.97	0.92	0.87	1.11
ACO2	0.53	0.60	0.77	0.38	0.52	0.59	0.57	0.63	0.76	1.20	0.95	0.85	1.19	0.94	0.85	1.01	1.15	0.93	0.99	1.06
ACSF2	0.62	0.61	0.50	0.48	0.42					1.17	1.08	1.03	1.06							
ACY1	0.31	0.48	0.32	0.16	0.23	0.20	0.36	0.29	0.37	1.05	1.47	0.82	0.90	0.89	1.02	0.86	1.11	0.92	0.81	1.06
AKR1A1	0.36	0.40	1.10	0.52	0.38	0.57	0.55	0.56	0.96	0.84	1.00	1.12	1.16	1.01	0.71	0.62	1.14	0.88	0.98	1.15
ALDH2	0.37		0.43	0.27	0.38	0.42	0.49	0.41	0.45	1.14	1.23	0.97	1.23	0.93	0.70	1.02	1.16	0.79	0.83	1.04
ALDH4A1			0.27	0.26	0.19	0.32		0.38	0.45	1.17	1.06	0.94	1.43	0.90	0.44	0.91	0.92	0.89	0.88	0.99
ALDH6A1	0.28	0.30	0.24	0.21	0.25	0.30	0.30	0.26	0.49	1.34	1.14	0.98	1.27	0.91	0.75	0.94	1.23	0.96	0.82	1.07
ALDOB	0.47	0.71	0.43	0.43	0.64	0.42	0.47	0.35	0.53	1.14	0.94	1.10	1.34	1.15	0.44	0.95	0.96	0.78	1.06	1.05
ASS1			0.19	0.15	0.18	0.17	0.31	0.19	0.37	1.16	1.12	0.80	1.14	0.89	0.42	0.81	0.87	0.80	1.01	1.10
ATP5A1			0.60	0.37	0.40	0.63	0.59	0.60	0.72	1.15		0.91	1.15	0.98	1.05	0.90	1.40	0.90	1.15	1.10
BDH2			0.56	0.50	0.37	0.48	0.55	0.39	0.89	0.94		1.27	0.89	0.94	0.89	0.78	1.08	0.85	0.82	1.13
BHMT		0.69	0.59	0.39	0.37	0.57		0.61		1.16	1.67	0.95	1.25	0.78	0.52			1.02	0.72	
CAT	0.57	0.61		0.41	0.82	0.40	0.52	0.47	0.73	1.09			1.17	0.89	0.96	0.95	0.86	1.13	0.94	0.99
CTSB			0.45	0.35		0.53	0.50	0.52		0.71		0.75	0.91	0.80	0.74	0.55	1.09	1.21	1.02	
CYCS	0.43		0.64	0.43		0.61	0.61	0.53	0.56	0.91	1.25	1.74	0.88	0.91	1.26	0.90	1.30	0.68	0.74	1.02
DDC			0.51	0.20	0.40	0.36	0.38	0.73	0.69	0.92	0.98	0.66	1.10	0.91	0.50	0.70	0.83	0.89	0.77	1.03
ECHS1			0.37	0.28		0.33	0.34	0.31	0.54	1.21	1.04	1.34	1.33	0.96	0.69	1.04	1.15	1.02	0.95	1.14
ETFB	0.63	0.42	0.26	0.18		0.41			0.60		1.18	0.72		0.97	0.77		1.06			1.09
FBP1			0.57	0.35	0.33			0.57	0.60	1.16		0.89	1.46	0.86	0.68	0.88	1.05	0.81	0.92	1.00
GATM			0.32	0.31	0.40	0.45	0.53	0.19	0.55	1.18		1.08	1.09	0.93	0.48	0.96	0.91	0.93	0.91	0.96
GOT2			0.56	0.32	0.39	0.46	0.80	0.50		1.07	0.81	1.31	1.09	0.97	1.04	0.93	1.18	0.89	0.87	
GPD1			0.47	0.19	0.23	0.40	0.44	0.51	0.61	1.12	1.07	0.72	1.19	1.05	0.54	0.94	0.96	1.12	1.08	0.96
HADH			0.32	0.28		0.37	0.40	0.37	0.61	1.27	1.16	0.99	1.19	0.95	0.85	0.96	1.09	0.88	0.90	1.03
HNRNPA2B1	0.44	0.65	0.29	0.49	0.41	0.59	0.43	0.37	0.47	0.80	0.94	0.76	0.87	1.11	1.35	1.29	1.02	1.11	1.04
IDH2	0.47	0.38	0.35	0.25	0.47	0.49	0.54	0.58	0.53	1.11	1.33	0.93	1.00	0.90	1.45	0.94	1.37	0.84	0.86	1.18
K4	0.59		0.46	0.36	0.57	0.45	0.47	0.75	0.65	1.16	1.18	0.74	1.25	1.18	0.59	1.02	1.32	0.99	1.18	1.09
KHK	0.48	0.60	0.89	0.56	0.57	0.62		0.61	1.05	1.11	1.02	0.87	1.14	1.05	0.63	0.94	1.05	0.94	0.89	0.99
LDHB	0.41	0.46	0.64	0.63	0.32	0.59	0.60	0.60	0.73	0.94	0.94	1.05	1.01	0.92	0.92	0.82	1.07	0.87	0.94	1.15
MDH2	0.64	0.50	0.67	0.33	0.39	0.54	0.59	0.51	0.59	1.08	1.06	1.20	1.12	0.94	1.06	0.88	1.07	0.90	0.77	0.98
PCK2	0.52	0.42	0.29	0.20		0.24	0.31	0.27	0.45	1.24	1.22	1.05	1.61	0.96	0.41	1.05	0.94	0.89	0.89	1.05
PRDX3			0.30	0.21	0.35	0.52	0.46	0.41	0.55	1.17	1.04	0.65	1.09	0.95	1.12	0.95	1.08	0.89	0.89	1.03
SELENBP1	0.68	0.50	0.50	0.32	0.36	0.42	0.38	0.72	1.07		0.95	0.94	0.93	0.98	0.98	1.23	1.01	0.84	1.04
SORD			0.54		0.34	0.30	0.31		0.32	0.99	1.08	0.95	1.16	0.87	0.69	0.86	1.12	0.96	0.91	0.92
SPD1	0.61	0.54	0.55	0.29	0.29	0.46	0.50	0.50	0.73	1.26	1.10	0.91	1.26	0.92	0.85	0.88	1.04	0.96	0.96	1.22
TAGLN	0.97		0.28	0.34	0.70	0.59	1.43	0.34	0.48	0.61	0.69	0.57	0.89	0.88	1.16	0.75	1.57	1.31	0.91	1.06
TP5B			0.65	0.39	0.58	0.62		0.51	0.88	1.23		0.62	1.25	0.96	1.17	0.95	1.31	0.84	0.97	1.03

**Footnote: C = cancer, N = normal.** Protein symbols were used according to UniProtKB. For a full name of protein and its accession number see **Supplementary Table 3**.

**Figure 1:**
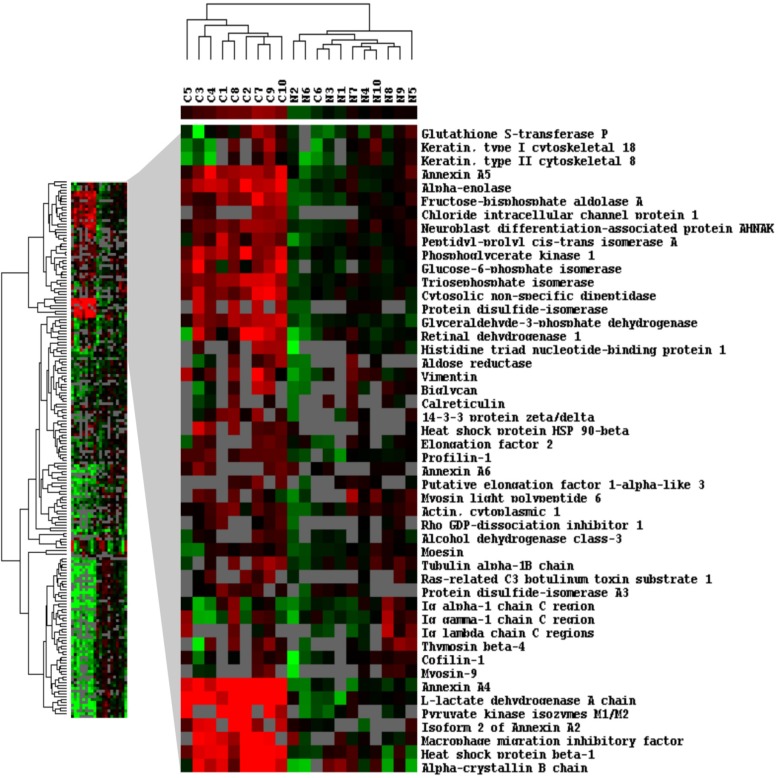
Hierarchical clustering analysis of dysregulated proteins between ccRCC and normal kidney tissues Clustering analysis was performed based on 345 proteins for which quantitative information was available. The samples clustered into two main groups: one that contained the 9 of 10 ccRCC samples (C1-C5, C7-C10); and second contained all normal samples (N1-N10) plus one cancer sample; C6. The difference in expressions of dysregulated proteins between ccRCC and normal kidney samples was statistically significant (p<0.001).

Unsupervised clustering was performed on the 55 significantly dysregulated proteins. The samples clustered into two main groups: one that contained 9 of 10 ccRCC samples; and second included all normal samples and one cancer sample (Figure 1). The difference in expressions of dysregulated proteins between ccRCC and normal kidney was statistically significant (p<0.001). To verify our analysis, we performed control clustering based on 345 quantified proteins excluding the 55 significantly dysregulated proteins. As expected, cancer and normal samples didn't cluster into distinct groups (p>0.5; Supplementary Figure 2).

### Identification of dysregulated proteins that can serve as serum biomarkers

Of 55 dysregulated proteins 39 (70.9%) satisfied one or more of the four criteria to be classified as “secretory” proteins (Supplementary Table 7); 5 proteins (9.1%) were found to be extracellular; 10 (18.2%) were membrane-bound; 8 (14.5%) could be released from cells via the exosome pathway; 1 (1.8%) is predicted to be classically secreted according to the SignalP analysis; and SecretomeP analyses predicted that 32 (58.2%) proteins are likely to be non-classically secreted.

**Table 2:** Involvement of dysregulated proteins in tumorigenesis-related processes

Tumorigenesis-related processes	Protein symbol
Carbohydrate and lipid metabolism	ACAA2, ACADM, ACAT1, AC02, ACSF2, ACY1, AKR1A1, ALDH2, ALDH4A1, ALDH6A1, ALDOA, ALDOB, ASS1, ATP5A1, BDH2, BHMT, CAT, CNDP2, DDC, ECHS1, ENO1, ETFB, FBP1, GAPDH, GATM, GOT2, GPD1, HADH, HSP27, IDH2, K4, KHK, LDHA, LDHB, MDH2, PCK2, PGK1, SORD, TP5B, TPI1
Apoptosis	ACAA2, ANXA2, ANXA4, ANXA5, CRYAB, CTSB, CYCS, ENO1, GAPDH, HSP27, HSPE1, LDHA, MIF, SELENBP1, SPD1
Growth and proliferation	CAT, ENO1, FBP1, HNRNPA2B1, HSP27, HSPE1, LDHA, MIF, PRDX3, SELENBP1, SPD1
Cell cycle	ENO1, HSP27, HSPE1, MIF
Hypoxia	ENO1, HSP27, LDHA

Protein symbols were used according to UniProtKB. For a full protein names and accession numbers, see Supplementary Table 3.

**Table 3:** Association between serum Hsp27 with clinical parameters in clear cell renal cell carcinoma patients

		Number of patients (%)		
Variables	n	Low Hsp27	High Hsp27	p-value
Status				
Normal	18	18 (42)	0(0)	0.008
ccRCC	36	25 (58)	11(100)	
Sex				
Male	23	15 (60)	8(73)	0.464
Female	13	10 (40)	3 (27)	
Tumor Grade				
Low (1–2)	26	15 (60)	11(100)	0.013
High (3–4)	10	10 (40)	0(0)	
Clinical Stage				
Low (1–2)	26	15 (60)	9(82)	0.393
High (3)	10	10 (40)	2(18)	

### Validation of the protein dysregulation

Of 39 “secretory” proteins we selected the 5 most promising for further verification as potential ccRCC biomarkers. The upregulated proteins ENO1 and Hsp27 were previously documented to be involved in tumorigenesis. AHNAK was a promising candidate with no record of its involvement in other malignancies. The downregulated HSPE1 was also documented to be involved in the pathogenesis of different malignancies.

**Figure 2:**
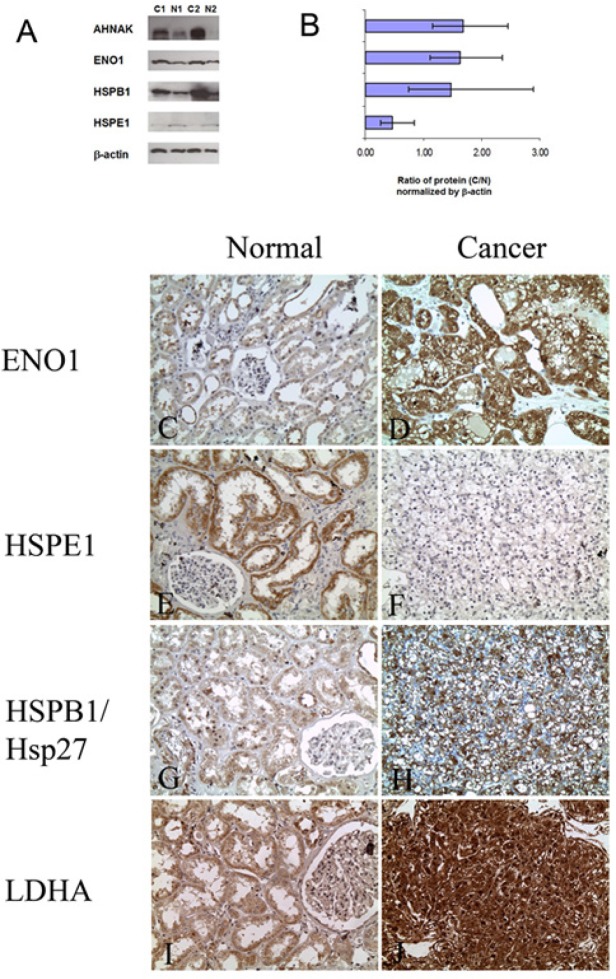
Verification of protein dysregulation in ccRCC by Western blot and immunohistochemical analyses A: Representative blots showing the expression of proteins in normal kidney tissues (N1, N2) and ccRCC (C1, C2). For AHNAK, ENO1, and HSP27, expression was significantly higher and for HSPE1 it was significantly lower in cancer compared to normal kidney tissue, β-actin was used as a loading control. B: Graphical representation of the average fold change in expression of the proteins between ten ccRCCs and matched normal specimens as determined by densitometry (C/N). Expressions of protein in normal samples were normalized. C-J: Representative photomicrographs showing differential expression of ENO1, HSPB1, HSPE1 and LDHA in ccRCC compared to normal kidney tissue by immunohistochemistry (Original magnification × 200).

In the first step, we verified differential expressions of these four proteins by Western blot analysis in samples of the discovery cohort (Figure 2A). The expressions of AHNAK, ENO1, and Hsp27 were found to be significantly elevated [1.68 fold (p<0.002), 1.62 fold (p<0.01), and 1.47 fold (p<0.01), respectively] in ccRCC compared to matched normal tissues. HSPE1 was significantly downregulated in ccRCC [0.47 fold (p<0.002)], in agreement with our MS findings (Figure 2B). Overall, these results confirm our MS analysis.

We additionally verified the dysregulation of ENO1, HSPB1, HSPE1, and LDHA in an independent cohort of patients by IHC using TMAs consisting of 85 cases of ccRCC and matched normal kidney tissue from the same patient (Figure 2C-J). We found that ENO1 immunoexpression was increased in 56 (70%) cases of ccRCC when compared to normal kidney tissue from the same patient (Supplementary Table 8), which was in agreement with both the MS and Western blot results.

When we examined the expression of HSPB1/Hsp27, 53 (69%) cases showed increased expression in ccRCC compared to normal matched tissues. 11 (14%) cases showed no significant change in expression between ccRCC and normal, and 13 (17%) cases showed decreased expression in ccRCC. The downregulation of HSPE1 was also seen immunohistochemically as 71 (91%) cases of ccRCC showed decreased expression compared to matched normal. Only 6 (8%) cases showed similar expression levels between normal and cancer. Comparable findings were seen for LDHA where there was increased expression in 76 (96%) ccRCC tissues examined. Only one case showed decreased expression in cancer and two cases (3%) showed no change in expression. The results of the IHC analyses are summarized in Supplementary Table 8.

### Elucidating RCC pathogenesis through quantitative proteomics

**Figure 3:**
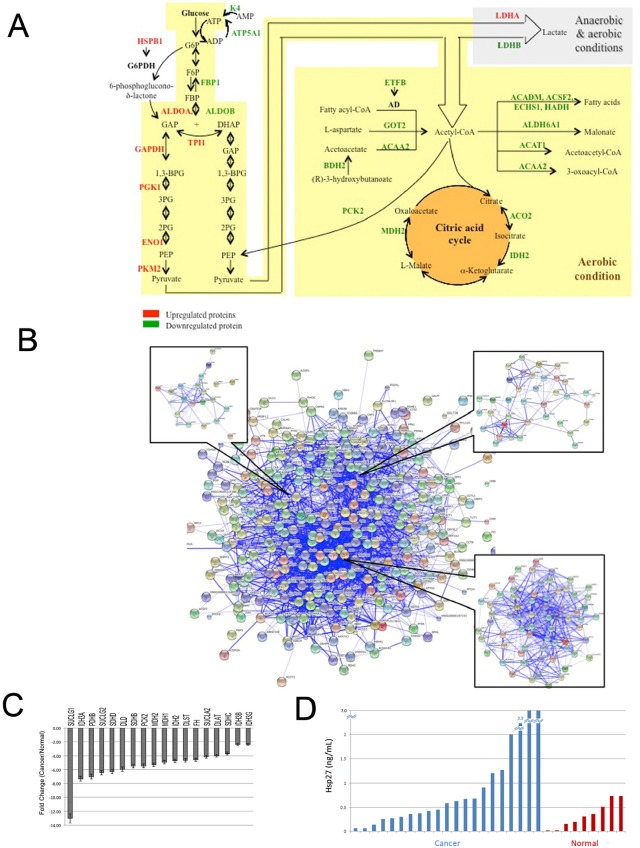
The involvement of dysregulated proteins in kidney cancer in metabolic pathways A: Most of the upregulated proteins are enzymes which catalyze the reactions of glycolysis, citric acid cycle, metabolism and catabolism of Acetyl-CoA. ETFB serves as a specific electron acceptor for several dehydrogenases, including five acytyl-CoA dehydrogenases (AD), glutaryl-CoA and sarcosine dehydrogenase; and HSP27 forms a complex with G6PDH that increased its activity. Upregulated proteins are shown in red and downregulated ones in green. B: Visualization of protein-protein interactions for dysregulated proteins in ccRCC using STRING analysis. Dysregulated proteins were used as input for STRING and are represented as spheres of distinct colors. Blue lines represent interactions between proteins and the thickness of the lines display the level of confidence associated with each interaction. Our dysregulated proteins formed one main cluster. Within this cluster, we identified three mini-clusters which contained proteins related to cell metabolism. C: PCR validation of the down-regulation of genes involved in the TCA cycle. We found that 18 of the 28 (64%) genes examined had significant decreased expression in ccRCC tissues when compared to normal kidney tissue. SUCLG1, p=0.003; IDH3A, p=0.026; PDHB, p<0.001; SUCLG2, p=0.006; SDHD, p=0.012; DLD, p=0.020; SDHB, p=0.007; PCK2, p=0.037; MDH2, p=0.043; MDH1, p<0.001; IDH2, p=0.030; DLST, p=0.037; FH, p=0.006; SUCLA2, p=0.001; DLAT, p<0.001; SDHC, p<0.001; IDH3B, p=0.006; IDH3G, p<0.001. D: Bar graph showing differential expression of Hsp27 in urine. We assayed the expression of Hsp27 by ELISA in 21 pre-operative RCC patients and 9 individuals with no malignancy. The average expression in RCC patients was significantly higher than those with no malignancy (1.822ng/mL vs. 0.3365ng/mL, respectively, p<0.05).

Next, we wanted to elucidate the role of these proteins in RCC pathogenesis and identify pathways of carcinogenesis that can have a diagnostic and/or therapeutic impact. We performed UniProtKB and literature searches on the 55 dysregulated proteins. All proteins, with the exception of AHNAK, were reported to be involved in at least one tumorigenesis-related process (Table 2 and Supplementary Table 7). We then performed Gene Ontology, pathway analysis and protein-protein interaction analyses. In agreement with recent reports [18–21], 40 dysregulated proteins were found to be involved in carbohydrate and lipid metabolism. 28 proteins are involved in glycolysis, citric cycle, and Acetyl-CoA metabolism, as shown in Figure 3A.

Fifteen proteins were found to be involved in apoptosis (Table 2 and Supplementary Table 7). For example, alpha-crystallin B chain, annexin A4, and macrophage migration inhibitory factor (MIF), among others negatively regulate the apoptotic process. Eleven proteins are involved in growth and proliferation. For instance, catalase promotes cell growth; LDHA forces pyruvic acid into the Krebs cycle rather than the glycolysis process, which inhibits cell growth. MIF negatively regulates cell cycle arrest. Also, silencing of ENO1 resulted in and cell cycle arrest of gastric cancer cells.

We also performed protein-protein interaction analysis and found significant protein-protein interactions among our dysregulated proteins. These proteins formed one main cluster (Figure 3B), suggesting the presence of many interactions within this protein set. We also identified three smaller clusters within the main cluster. All three mini-clusters were related to glucose metabolism. In particular, we found the largest of the mini-clusters contained proteins that are involved in the TCA cycle.

### Experimental validation of metabolic dysregulation in ccRCC

We confirmed downregulation of genes involved in the citric acid cycle by PCR (Figure 3C). Using PCR arrays, we found 18 of the 28 genes (64%) involved in the citric acid cycle showed significant decreased expression in ccRCC tissues when compared to normal kidney tissue. The most downregulated genes were succinate-CoA ligase (SUCLG1, −13.0 times fold change, p=0.003) and isocitrate dehydrogenase 3 (NAD+) alpha (IDH3A, −7.31 times fold change, p=0.026). Another gene that showed a high degree of downregulation in ccRCC tissues was pyruvate dehydrogenase (PHDB,, −7.0 times fold change, p<0.001).

### Hsp27 is a potential biomarker in RCC

We performed a preliminary analysis to evaluate the potential utility of Hsp27 and ENO1 as serum and urine markers in ccRCC. We found significant differences in the levels of urinary Hsp27 between patients with ccRCC compared to normal individuals (average 1.822ng/mL vs. 0.3365ng/mL, respectively (p<0.05; Figure 3D). These data suggest Hsp27 may be a useful diagnostic marker for RCC patients.

Serum analysis showed that average Hsp27 expression in patients with ccRCC was 2.334ng/mL compared to 1.233ng/mL in non-cancerous individuals. Using a cut-off of 2.529ng/mL (average Hsp27 expression of non-cancerous patient's ± 2 SD) patients were classified as having high or low Hsp27 expression. Patients with high Hsp27 were more likely to have ccRCC (p=0.008; Table 3). Also, higher tumor grades (grade 3–4) were associated with higher Hsp27 expression (p=0.013).

We also examined the expression levels of ENO1 in serum and urine and found that expression was higher in ccRCC compared to normal but the differences did not reach statistical significance. The average expression of ENO1 in the serum of RCC patients and patients with no malignancy was 0.0061ng/mL and 0.0057ng/mL, respectively. Urine samples had 0.007187 ng/mL vs. 0.006842 ng/mL, respectively (data not shown).

## DISCUSSION

A significant challenge in ccRCC is the lack of tools that can distinguish between benign and malignant kidney masses. Imaging has limited accuracy and cannot be used reliably to confirm the nature of the lesion. This results in some patients having their kidneys unnecessarily removed for benign lesions. Biopsy is an invasive procedure with limited success and a number of side effects. A non-invasive urine or blood-based test will represent a revolutionary step in RCC diagnosis. We performed comprehensive quantitative proteomics analysis and identified promising biomarkers that can be further investigated as non-invasive tests for the accurate diagnosis of kidney cancer. We identified 55 proteins that are significantly dysregulated in ccRCC relative to normal kidney tissues (Table 1). Hierarchical clustering showed the ability of these proteins to distinguish between RCC and normal kidney tissues (Figure 1). Our results are in agreement with previous reports[9;18;19;22–26].

There are a number of unique aspects in our study. The two-dimensional liquid chromatographic approach employed is considered superior to 2D-PAGE. Proteins with molecular masses higher than 150 kDa and lower than 15 kDa as well as proteins with isoelectric points outside the range of pH 3 — 10 cannot be identified by 2D-gels and hydrophobic membrane proteins are underrepresented on 2D gels. Advanced features of our mass spectrometric analyses include simultaneous profiling of multiple admixed specimens which helps to eliminate artifactual differences due to differential protein losses during purification or separation. In addition, the use of four isotopic labels allows quantitative comparison among four different tissue samples.

Of 55 dysregulated proteins, 39 (70.9%) were classified as “secretory” proteins and thus have the potential to serve as serum/urine diagnostic biomarker for ccRCC. Interestingly, ENO1, LDHA, and Hsp27, AHNAK have been reported in other malignancies to be upregulated in serum of cancer patients, compared to healthy controls: serum ENO1 was elevated in patients with small-cell lung carcinoma; and high serum LDH was linked with significantly poor survival of colorectal cancer[27]; high level of Hsp27 was present in serum from patients with breast cancer[28]; and serum AHNAK level was elevated in patients with ovarian cancer[29]. That suggests that upregulation of these proteins can be detected in serum of ccRCC patients as well. Our pilot study shows a potential of Hsp27 as a serum/urine biomarker. This needs to be validated in a larger cohort of patients.

Our results also strongly suggest that ccRCCs have a protein “signature” that is required for carcinogenesis. All 55 dysregulated proteins had previously been reported to be involved in tumorigenesis processes. Interestingly, the majority of these proteins are involved in “metabolic” processes (Figure 3A and Supplementary Table 7). Dysregulated cellular metabolism is adapted by cancer cells to meet the requirements of rapid cell proliferation, growth, negative regulation of apoptosis, survival under hypoxia etc.

Our findings are comparable to earlier reports on functional analyses in ccRCC[30;31]. The link between carbohydrate metabolism and RCC is not surprising. In contrast to normal proliferating cells, tumor cells have to survive in environments with varying oxygen and nutrient supplies [32]. The increase in lactate dehydrogenase and the activation of the pyruvate kinase pathways indicate active anaerobic glycolysis which is a reflection of the hypoxic conditions known to be an integral component of the pathogenesis of RCC[33;34]. Also the “clear cell” morphology of RCC is known to result from the accumulation of glycogen as a result of disturbed carbohydrate metabolism.

Global analysis through molecular profiling and pathway analysis can have significant clinical applications. In addition to identified new therapeutic targets, pathway-derived metabolic products can be also used as diagnostic, predictive and prognostic markers [35].

In conclusion, through quantitative proteomic analysis, we identified differential protein expressions that can distinguish between ccRCC and normal kidney tissues. Most of these proteins are involved in biological pathways pertinent to carcinogenesis. About 70% of these proteins can be potentially shed into serum. If the upregulation of the most promising proteins would be confirmed in serum/urine of ccRCC patients, then that would suggest their potential as noninvasive biomarkers for confirming the diagnosis of RCC, which may greatly improve patient management and increase overall survival.

## MATERIALS AND METHODS

### Specimen preparation and protein extraction

This study included a total of 199 patients. For the discovery set, 40 samples (normal and cancerous tissues) were used. For the first validation set, we analyzed 85 cases with matching normal and cancerous tissue from the same patient. Serum analysis was done using 54 preoperative samples of RCC and 36 normal individuals. Urine analysis was done on 9 normal persons compared to 21 pre-operative urines from ccRCC patients.

ccRCC and matching normal kidney tissues from the same patient were obtained from nephrectomy specimens at St. Michael's Hospital, Toronto, Canada within 15 min post-surgery and snap frozen in liquid nitrogen. As ccRCC is known to arise from the proximal tubules[8], the kidney cortex is considered a suitable representation of normal kidney [9]. All specimens were histologically confirmed. The study was approved by the Research Ethics Board of St. Michael's Hospital. Relevant clinical information on the patients is shown in Supplementary Table 1.

Tissues were prepared as described previously[10;11]. Briefly, tissues were homogenized in a protease-inhibitor cocktail (Roche). Cell debris was separated and clear supernatant was used for analysis. A reference sample was prepared from a pool of 30 normal kidney tissues. Protein concentrations were determined using the Bradford assay (Sigma-Aldrich, St. Louis, USA) [11;12].

### iTRAQ sample labeling and strong cation exchange (SCX) chromatography

For iTRAQ LC-MS analysis, 100 μg of sample were denatured, disulfide bonds were reduced, and the cysteine residues were blocked as per the iTRAQ protocol (Applied Biosystems, Foster City, CA) and digested with trypsin. Samples were then labeled with the iTRAQ tags (Supplementary Table 2). Labeling of the reference sample was randomized for each set to eliminate any potential bias associated with a particular iTRAQ reporter tag. Samples were then dried using a vacuum centrifuge.

The iTRAQ sets were dissolved in 1.7 mL of Buffer A (Supplementary Table 3) and filtered using a 0.45-μm syringe filter (Millipore, Cambridge, ON, Canada). Each set was then separated by off-line SCX chromatography using an HP1050 HPLC instrument (Agilent, Palo Alto, CA). Separation was performed using a linear binary gradient over 1 h. Buffer C was used to strip the column after the run. A total of 30 SCX fractions were collected per iTRAQ set. These fractions were dried using a vacuum centrifuge.

### Reverse phase (RP) LC-MS

The SCX fractions were analyzed in triplicate using a Nanobore LC system (LC Packings, Amsterdam. Netherlands) and a QSTAR Pulsar mass spectrometer (Applied Biosystems/MDS SCIEX, Foster City, CA) in positive ion mode. Fractions were re-dissolved in 16 μL of eluant A [94.9% deionized water, 5.0% methanol, and 0.1% formic acid (pH 3)]. For subsequent fractions, the amount of eluant A was incremented by 2 μL over the preceding fraction to accommodate the increase in the amount of KCl. A 1-μL aliquot of the sample (~1 μg of total peptides) was loaded onto a C18 RP pre-column (LC Packings: 300 μm × 5 mm) and desalted before separation on an RP analytical column (75-μm × 150-mm packed in-house with 3-μm Kromasil C18 beads with 100 Å pores, The Nest Group, Southborough, USA). Eluant A was used to load the sample onto the C18 pre-column at a flow rate of 25 μL min^−1^. After 4 min, the C18 pre-column was switched in-line with the RP analytical column. Separation was performed at 100 nL min^−1^ using a nonlinear binary gradient (Supplementary Table 4) starting with eluant A and transitioning to eluant B (5.0% deionized water, 94.9% methanol, and 0.1% formic acid).

MS data were acquired in information-dependent acquisition (IDA) mode using the Analyst QS 1.1 software (Applied Biosystems/MDS SCIEX). The LC-MS analysis was performed using a 1-s TOF-MS survey scan from 400 to 1500 Da, followed by four, 2-s product-ion scans, from 80 to 2000 Da, of the four most-abundant ion peaks in the survey scan. The collision energy (CE) was automatically controlled by the IDA CE parameter script. Switching criteria were set for ions with *mlz* ≥ 400 and <1500, charge states of +2 to +4, and abundances of ≥10 counts. Using Analyst QS 1.1 controlled dynamic exclusion, former target ions were excluded for 30 s, and ions within a 100-ppm window were ignored. Precursor ion exclusion lists were used to minimize redundancy.

### Bioinformatics Analysis

#### Protein identification by Protein Pilot

MS data of each fraction was used to identify proteins by searching a concatenated Swissprot/Panther database of 66082 distinct human protein entries (version June 2, 2010). The database was searched using Protein Pilot software, version 2.0.1 (AB SCIEX, Foster City, USA), which uses the Paragon algorithm[13]. Protein identification was performed at a confidence threshold of 95% (Protein Pilot Unused score ≥1.3) with MMTS selected as cysteine modification, with the search option ‘emphasis on biological modifications' checked, and with one of missed and/or non-specific cleavages permitted. Peptide and protein summaries were generated.

To minimize redundancy in subsequent iteration, a precursor ion exclusion list, generated in-house, was added to the acquisition method after each iteration as described previously [14]. Tolerance windows for exclusion were set at 100 ppm for *m/z* and 360 s for elution time.

### iTRAQ ratio re-calculation and identification of dysregulated proteins

To identify non-redundant proteins, data acquired for all 25 fractions from each iTRAQ set injected in triplicate were searched against a database that was created by concatenating the Swissprot human protein database and its reverse (as of June 2, 2010). Only proteins identified with local false discovery rate (FDR) ≤ 5% were considered for further analysis[15].

Proteins identified in seven iTRAQ sets were compiled and matched by accession numbers. Redundant proteins and peptides, and proteins identified in reverse sequence were removed from the list. To improve the confidence of protein quantitation, the mean expression iTRAQ ratios of the proteins were re-calculated, using a script written in Matlab (version 7.7.0.471), based on the criteria that the protein must be identified by a minimum of three peptides, with ≥95% confidence, and with an expression ratio error factor (EF) <11.1%. To enhance confidence in the protein quantitation even more, we included only 95% of all quantified proteins with the lowest computed EF (which corresponds to a confidence > 0.05 in Supplementary Table 5) for further consideration. Proteins were considered to be dysregulated if iTRAQ ratios were ≥1.5 or ≤0.67 in ≥50% in ccRCC relative to reference samples.

### Clustering analysis of dysregulated proteins in ccRCC samples

Proteins were included in the analysis if quantification was available in at least 50% of the samples. The average iTRAQ ratios were logarithmically transformed, for hierarchical clustering was used the City-block distance method. As a control, the samples were hierarchically clustered based on quantified proteins without dysregulated proteins. Hierarchical clustering analysis was performed using the Cluster 3.0 software and the result was visualized with the TreeView software [16]. To assess the statistical significance of the difference between proteins expressions in ccRCC and normal kidney samples were used the Welch's two-tailed t-test. Value of p<0.05 was considered as significant.

### Selection of candidate ccRCC markers

Dysregulated proteins were selected for further verification if they had a documented role in tumorigenesis and a potential to be identified in serum. We classified a dysregulated protein as “secreted” if it satisfied at least one of the following four criteria: (1) its subcellular location is extracellular or membrane-bound; (2) it is non-classically secreted by the exosome pathway, (3) it is classically secreted, according to SignalP 4.0 analysis; or (4) it is non-classically secreted, according to SecretomeP 2.0 analysis.

### Pathway and protein-protein interaction analysis

Dysregulated proteins were subjected to pathway analysis using Reactome and KEGG software. Predicted protein-protein interactions were generated and visualized using STRING 9.0 software [17]. Parameters used for species and confidence were, “homo sapiens” and “medium confidence (0.400),” respectively. Clusters of proteins were determined through confidence levels of surrounding protein-protein interactions.

### Western Blot analysis

20μg of total protein were separated on a 10% SDS-PAGE gel, transferred to a PVDF membrane, and probed with rabbit polyclonal antibodies to L-lactate-dehydrogenase A (LDHA) and α-enolase (ENO1), and mouse monoclonal antibodies to 10kDA heat shock protein (HSPE1), neuroblast differentiation-associated protein (AHNAK), heat shock protein β1 (HSPB1/Hsp27), and β-actin (Abcam, Cambridge, USA) overnight at 4°C. Intensity of protein staining was determined using ImageJ (http://rsbweb.nih.gov/ij/). Tumor samples were compared to normal kidney samples using the paired sample two-tailed t-test. Value p<0.05 was considered as significant.

### Tissue microarray construction and IHC

Tissue microarray (TMA) blocks were constructed as described previously [14] and contained 85 cases of ccRCC and matched normal kidney tissue from the same patient. Sections were deparaffinized, hydrated in ethanol, pre-treated in a microwave oven for 20 min at 800 W in 1 L of citrate buffer (0.01 M, pH 6.0), and incubated with hydrogen peroxide (0.3% v/v) in PBS for 15 min. Sections were blocked with 10% fetal bovine serum and incubated with primary antibodies for Hsp27, ENO1, LDHA, and HSPE1 overnight at 4°C. Protein expression was detected with Dako LSAB+ kit (Dako Cytomation) and counterstained with Mayer's hematoxylin.

Immunoexpression was scored by assessing the cytoplasmic, nuclear, and membrane staining intensity and frequency. Intensity was scored as 0 (no expression); 1 (weak); 2 (moderate); and 3 (strong). Frequency was scored as 0 (no expression); 1 (1–25%); 2 (26–50%); 3 (51–75%); and 4 (76–100%). Cancer scores that had a combined sum that was ±1 from the normal score were not considered significant and were called “no change.”

### ELISA

Human ELISA kits were purchased for Hsp27 and ENO1 (AbCam) and procedures were performed as recommended by the manufacturer. Urine samples were obtained from 11 patients with primary ccRCC and six patients with no malignancy. Serum samples were analyzed for a total of 21 patients with ccRCC and 9 patients with no malignancy.

### RT2 Glucose Metabolism Profiler Assay

We used the Human Glucose Metabolism RT^2^ Profiler™ PCR Array (SA Biosceinces) to examine the expression of 84 key genes involved in the regulation and enzymatic pathways of glucose and glycogen metabolism. Total RNA was extracted using the RNeasy Kit (Qiagen) and RNA integrity was assessed using the Agilent 2100 Bioanalyzer (Agilent). Four matched pairs of tumor and normal matched kidney tissue from the same patient were analyzed (a total of eight samples). Briefly, cDNA was synthesized from 1 μg total RNA using the RT^2^ First Strand Kit (SA Biosceinces) according to the manufacture's protocol. Reaction mixtures were incubated at 42°C for 15 minutes, 95°C for 5 minutes and held on ice until the PCR reaction. PCR cycling conditions were as follows: 95°C for 10 min, followed by 40 cycles of 95°C for 15 sec and 60°C for one minute, on the Step One Plus (Life Technologies). Relative quantification Changes in expression were measured by obtaining the threshold cycle and normalizing to the average of three housekeeping genes including Hypoxanthine Phosphoribosyltransferase 1 (HPRT1), Ribosomal Protein L13a (RPL13A), and β-actin (ACTB). Fold change was calculated by 2^(-ΔΔCt)^ where ΔΔC_t_ = [CAKI-1 cells transfected with miR-215 (C_t_ target-C_t_ control)] - [untransfected CAKI-1 cells (C_t_ target-C_t_ control)].

## Supplementary Figures




