# Supplementary figures and images for: Quantitative proteomic analysis reveals potential diagnostic markers and pathways involved in pathogenesis of renal cell carcinoma
Source: Oncotarget. 2014 Jan 18;5(2):506–18. doi: 10.18632/oncotarget.1529 (PMC3964225; doi:10.18632/oncotarget.1529)

Supplementary  
Figure 1

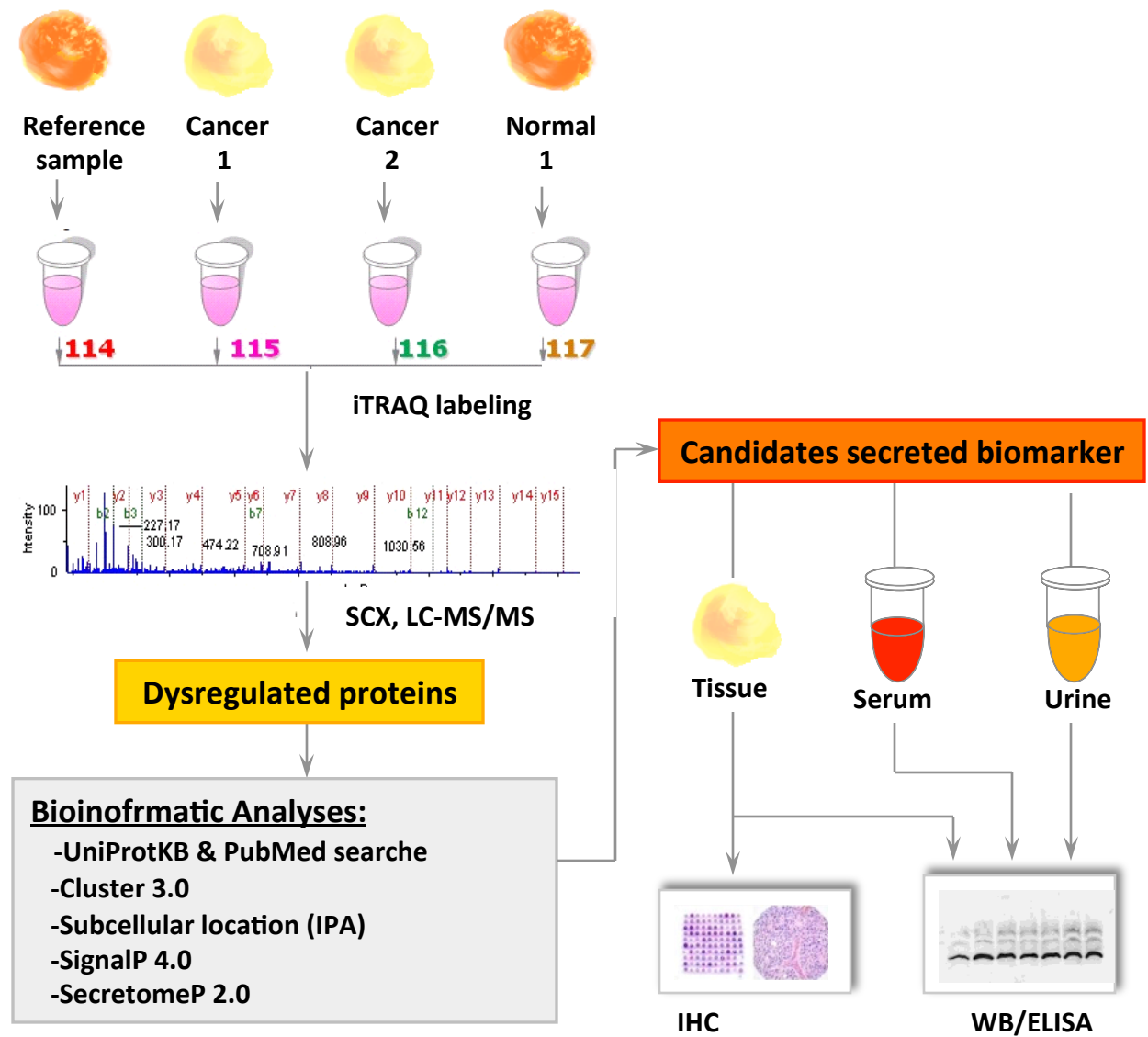

Supplement: Supplementary file 1 [file oncotarget-05-506-s001.pdf]

Supplementary Figure 2

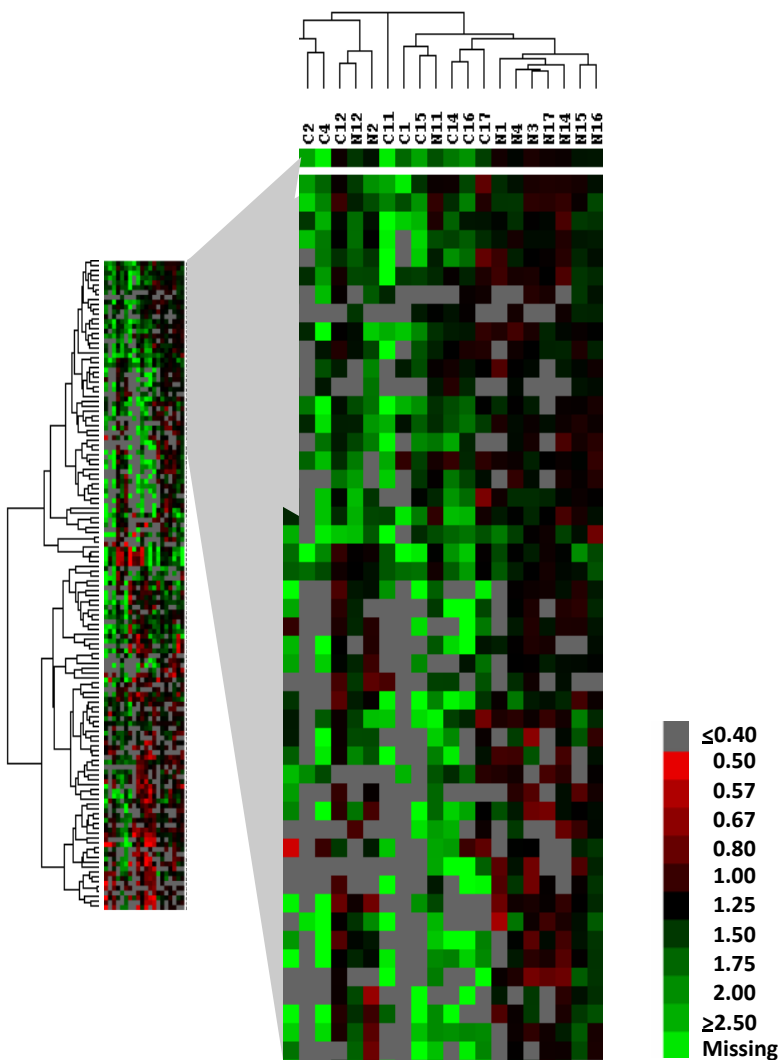

Supplement: Supplementary file 2 [file oncotarget-05-506-s002.pdf]
